# Blanking on blanks: few insect microbiota studies control for contaminants

**DOI:** 10.1128/mbio.02658-24

**Published:** 2025-02-25

**Authors:** Elisabeth M. Williamson, Tobin J. Hammer, Katja Hogendoorn, Raphael Eisenhofer

**Affiliations:** 1 School of Agriculture, Food and Wine, The University of Adelaide, Adelaide, Australia; 2 Department of Ecology and Evolutionary Biology, University of California, Irvine, California, USA; 3 Centre for Evolutionary Hologenomics, Globe Institute, The University of Copenhagen, Copenhagen, Denmark; Max Planck Institute for Chemical Ecology, Jena, Germany

**Keywords:** microbiome, symbionts, bacteria, kitome, 16S, contamination

## Abstract

Research on insect-microbe relationships is booming, with DNA sequencing being the most commonly used method to describe insect microbiota. However, sequencing is vulnerable to contamination, especially when the sample has low microbial biomass. Such low-biomass samples are common across insect taxa, developmental stages, and tissue types. Identifying putative contaminants is essential to distinguish between true microbiota and introduced contaminant DNA. It is therefore important that studies control for contamination, but how often this is done is unknown. To investigate the status quo of contamination control, we undertook a systematic literature review to quantify the prevalence of negative control usage and contamination control across the literature on insect microbiota (specifically bacterial communities) over a 10 year period. Two-thirds of the 243 insect microbiota studies evaluated had not included blanks (negative controls), and only 13.6% of the studies sequenced these blanks and controlled for contamination in their samples. Our findings highlight a major lack of contamination control in the field of insect microbiota research. This result suggests that a number of microbes reported in the literature may be contaminants as opposed to insect-associated microbiota and that more rigorous contamination control is needed to improve research reliability, validity, and reproducibility. Based on our findings, we recommend the previously developed guidelines outlined in the RIDE checklist, with the addition of one more guideline. We refer to this as the RIDES checklist, which stands for Report methodology, Include negative controls, Determine the level of contamination, Explore contamination downstream, and State the amount of off-target amplification.

**IMPORTANCE:**

Our systematic review reveals a major lack of methodological rigor within the field of research on insect-associated microbiota. The small percentage of studies that control for contamination suggests that an unknown but potentially considerable number of bacteria reported in the literature could be contaminants. The implication of this finding is that true microbiota may be masked or misrepresented, especially in insects with low microbial biomass.

## INTRODUCTION

Research on insect-associated microbial communities is booming globally. Communities of microbial symbionts (microbiota) can have profound ecological and evolutionary impacts on insects, which in some cases form obligate, species-specific symbiotic relationships with their host (e.g., [1–3]). However, most insect microbiota are yet to be described, or are in early stages of investigation, and the extent of microbial reliance and the nature of these associations across the insect world are unknown. The most commonly used method for characterizing insect microbiota is DNA amplicon sequencing (4). While DNA sequencing is a highly powerful, sensitive, and accessible tool, interpretation of the data requires great care.

One of the biggest limitations of amplicon sequencing assessments is DNA contamination. Biological samples can become contaminated from exposure during collection, contact with research personnel and the laboratory environment, as well as from reagents, DNA extraction kits (called “kitomes”), and cross contamination between samples (called “splashomes”) (5–8). When uncontrolled, DNA contamination can result in erroneous community assessments by distorting taxonomic diversity, obscuring differences between samples, and misrepresenting true absences of microbiota (9). For example, human placental tissue was once thought to harbor specific microbiota, but later studies found that almost all of the sequence data could be attributed to contamination (6, 10, 11).

Contamination is particularly problematic when biological samples have a low number of microbes (called “low-biomass samples”). Low-biomass samples occur when microbes naturally exist in low abundance or because the biomass of the sample itself is small. These can include (but are not limited to) certain types of insects (12), mammalian tissues (13, 14), glacial ice (15), rocks (16), air (17), and man-made environments (18). Low-biomass samples are more prone to DNA contamination because there are fewer “true” microbes to crowd out the contaminants (5, 19), unlike in high-biomass samples where the issue of contamination is relatively minor. An empirical example of the relationship between low biomass and high contamination is given in Fig. S1.

Universal primers designed to amplify sequences of bacterial 16S rRNA genes can also amplify chloroplast and mitochondrial genomes (referred to as “off-target DNA”) due to their common evolutionary history (20, 21). Consequently, 16S sequencing assessments can experience amplification bias toward off-target DNA if samples are low biomass and contain animal or plant tissue. Chloroplasts and mitochondria are predominant in insect microbiota due to insect diets and/or tissues; however, off-target DNA can also be an issue in other contexts, such as in plant, human microbiome, or food-web studies (22–24). A high relative abundance of off-target DNA can distort and obscure community assessments by reducing the representation of low-abundance taxa, and this can further exacerbate the impact of contamination. Furthermore, there are several cases where high proportions of off-target DNA in insect samples are predicted to be the result of low microbial biomass, such as in *Lepidoptera* (12, 25), *Hymenoptera* (26), *Thysanoptera* (26), and *Phasmatodea* (27).

**Fig 1:**
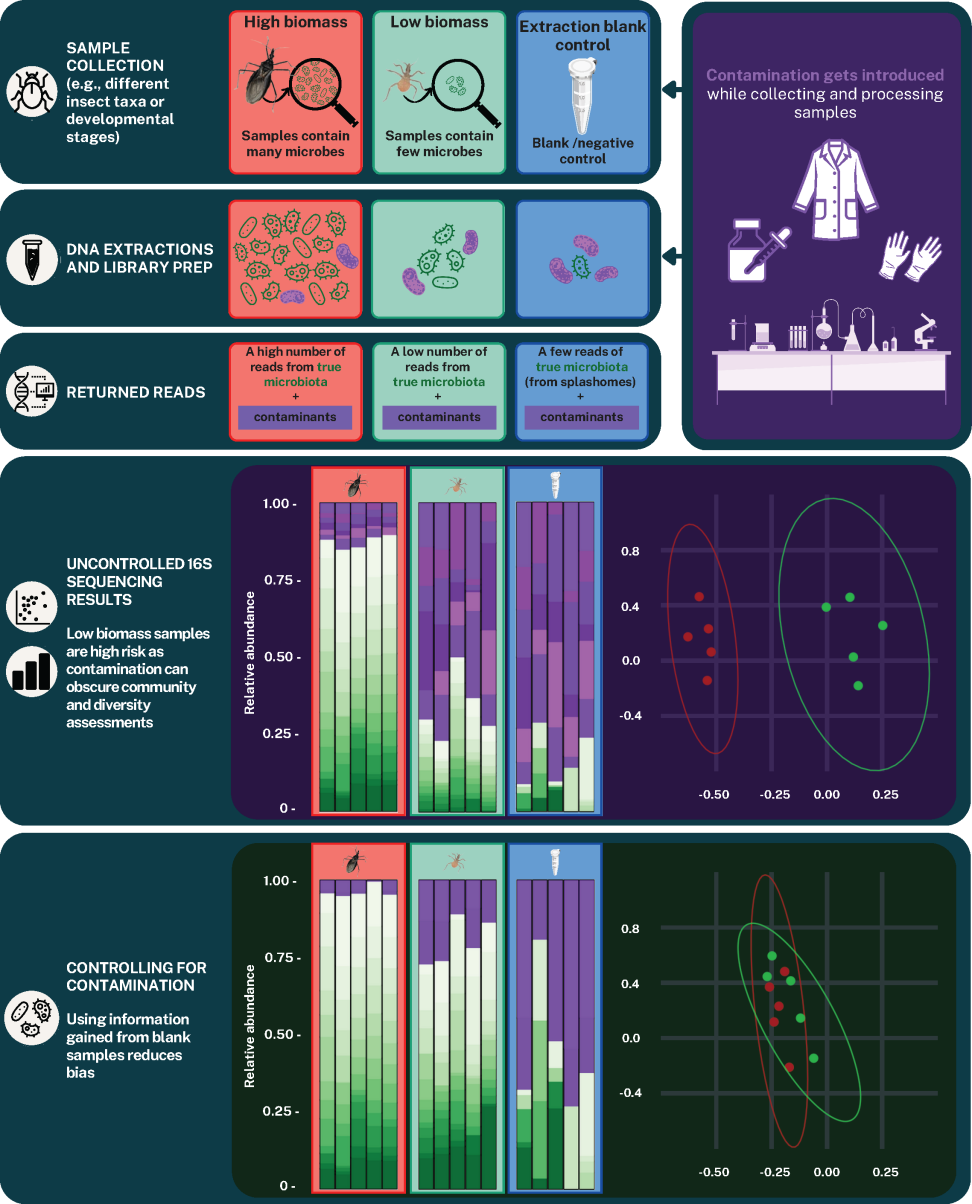
Conceptual schematic to demonstrate the different impact contamination (represented in purple) can have on high- vs low-biomass samples. Using relative abundance plots and principal coordinate analysis (PCoA) diversity assessments, uncontrolled contamination is shown to mask true taxa and similarities between samples. While not being perfect, due to differences in contaminant prevalence across samples, and/or splashomes, contamination control using sequence information from blanks reduces this bias.

There are established protocols to control for DNA contamination. The standard approach, and the method focused on in this systematic review, is to process no-template or negative controls (i.e., blank samples) alongside biological samples during DNA extraction, PCR, and sequencing. Amplicon sequence variants (ASVs) can then be removed from the biological sample data if they are detected and more prevalent in negative controls (5, 8, 19; Fig. 1). It is recommended to include a negative control per batch of DNA extractions, as the degree of contamination can vary between lots processed (28). The accuracy of such techniques can be further improved by employing statistical packages, such as Decontam (9), that classify ASVs as contaminants using established and reproducible methods. Another benefit to using statistical packages is that the method can be tailored according to the data set; for instance, Decontam provides the option to adjust the prevalence method used and modify the sensitivity of contamination identification (9).

Negative controls can also be used to measure the limit of detection (LoD). The LoD is a benchmark to determine the lowest amount of sample-derived DNA that can be reliably used to identify and quantify microbial taxa in a given data set (5, 29). The LoD can be measured using quantitative PCRs (qPCRs), where the absolute abundances in all samples and negative controls are measured. The average abundance in negative controls is used as the LoD, and anything above this is inferred to be amplified DNA from biological samples. If a biological sample falls below the LoD, it should be discarded as it does not meet the minimum threshold of “true” DNA (5, 19, 28).

Controlling for contamination is essential, but how often it is actually done in the insect microbiota literature is unknown. Here, we systematically assess whether insect microbiota research over a 10 year period has appropriately controlled for DNA contamination. Specifically, we address the following questions: (1) What percentage of studies have used negative controls to control contamination? (2) Has there been an increase in the proportion of studies that control for contamination over the years? (3) How many studies have determined their experimental limit of detection? (4) What proportion of studies acknowledge off-target amplification from chloroplast or mitochondrial DNA? In light of our findings, we provide recommendations to improve the robustness of future insect microbiota research.

## MATERIALS AND METHODS

### Search strategy, KAPPA analysis, and filtering

**Fig 2:**
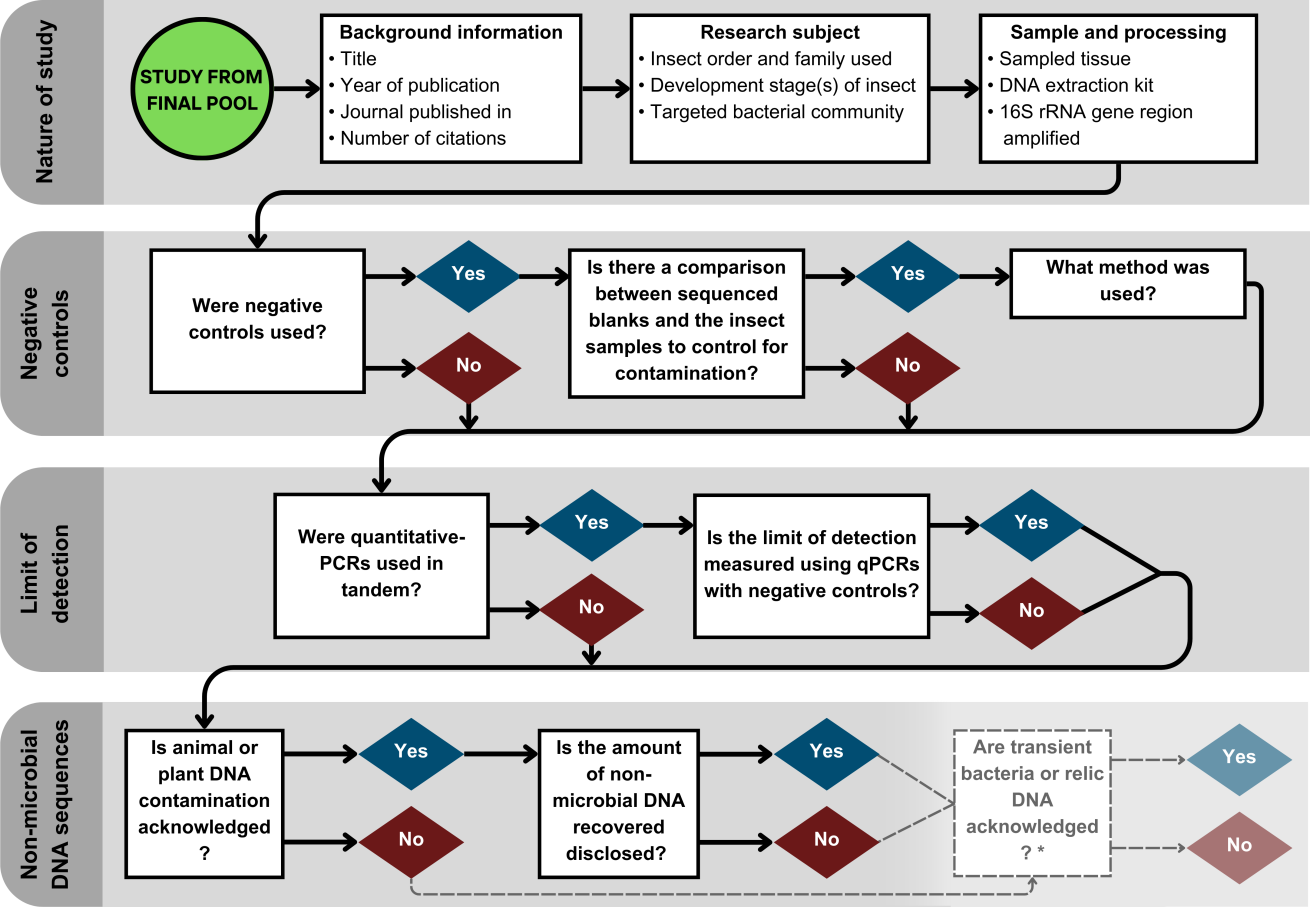
The information mined from the final pool of papers that researched insect microbiota using 16S sequencing to create the metadata set. * indicates additional questions addressed in the supplementary material.

This review was conducted according to the Preferred Reporting Items for Systematic Reviews and Meta-analyses Statement (PRISMA) (30). We systematically searched Scopus, Web of Science, and Google Scholar for relevant articles and data papers. Due to the lack of standardization between electronic databases, we tailored our search strategy for each database to include certain terms and synonyms relating to our key concepts. Specifically, we searched for papers that described or investigated the microbiota of insects (searches provided in supplementary materials, Fig. S2). Ordered by “relevance,” the first 200 search results from the three database searches were then imported into the Covidence platform (31), and duplicate papers were removed. Before filtering all imported studies using the Inclusion/Exclusion criteria (Table S1), 50 papers (sorted by “Title”) were assessed individually by two authors for comparison using a KAPPA analysis. Because the KAPPA analysis confirmed that the screening was reproducible, we proceeded with the remaining articles to produce the final pool of papers for metadata collection. A summary of the information gathered from our final pool is given in Fig. 2, and the detailed data extraction method is given in the supplementary materials (Table S2; Fig. S3 and S4).

### Metadata analysis

We first assessed the percentage of the most represented insect orders, to verify a lack of bias to certain taxa. We then examined trends in specimen sampling and processing by obtaining the percentages of the developmental stage(s) investigated (only adults, only juveniles, or multiple stages), the inclusion of a surface sterilization step, and the region of the 16S rRNA gene used. It was determined how many studies mentioned mitochondria and/or chloroplasts in their methods or results sections and, if mentioned, whether they disclosed the amount of off-target amplification. We calculated the proportion of studies that reported using a negative control and that used qPCRs to measure the limit of detection. From the subset of studies that used a negative control, we further calculated the proportion of studies that sequenced their controls and used this information to control for contamination. After assessing the distribution of citation counts using a Shapiro-Wilk test for normality, we opted to use a Mann-Whitney-Wilcoxon test to assess whether the number of citations differed between studies that did or did not control for contamination, as an indicator of their perceived usefulness.

## RESULTS AND DISCUSSION

### Exploring the metadata

Our final pool consisted of 243 papers published between 2011 and 2022, which were used for metadata collection and subsequent analyses (Fig. S4).

#### Representation of insect orders and sampling approaches

The top five insect orders represented in our final pool were *Diptera* (23%), *Hemiptera* (18.1%), *Lepidoptera* (16.9%), *Coleoptera* (14.8%), and *Hymenoptera* (14%). The remaining 13.2% studied *Blattodea*, *Odonata*, *Orthoptera*, *Psocodea*, *Siphonaptera*, or multiple different insect orders. The distribution of insect orders studied indicates that there was no bias to one field in our metadata. For the insects studied, 77.5% of studies sampled one developmental stage, with 51.4% of papers focusing on the adult stage, 25.9% sampling only juveniles (e.g., eggs, larvae, nymphs, or pupae), and the remaining 22.6% investigating more than one stage of development. A total of 56.8% of studies aimed to describe the microbial community of the gut specifically, 39.1% of studies described the community for the whole insect, and the remaining targeted specific organs or tissues.

#### Trends in specimen processing methods

A total of 66.7% of studies included a surface sterilization step. A range of substances were used to wash insects, with ethanol, bleach, sterile water, PBS, and detergent most frequently used. The variation in chemicals and whether or not insects were washed exemplifies the current lack of uniformity throughout insect-microbiota studies. In addition, many papers that included a surface sterilization step stated that the purpose of the procedure was to remove external microbes. Contaminating microbes on the surface of specimens can be introduced during handling and storage, or occur naturally as a consequence of environmental contact (i.e., transient bacteria). It is possible that in some cases, surface sterilization was presumed sufficient to control contamination. However, this fails to acknowledge downstream contaminants introduced during molecular processing, and previous research has shown no detectable effect of surface sterilization on insect microbiota (32).

### Prevalence of negative control usage and controlling contamination in insect microbiota studies

**Fig 3:**
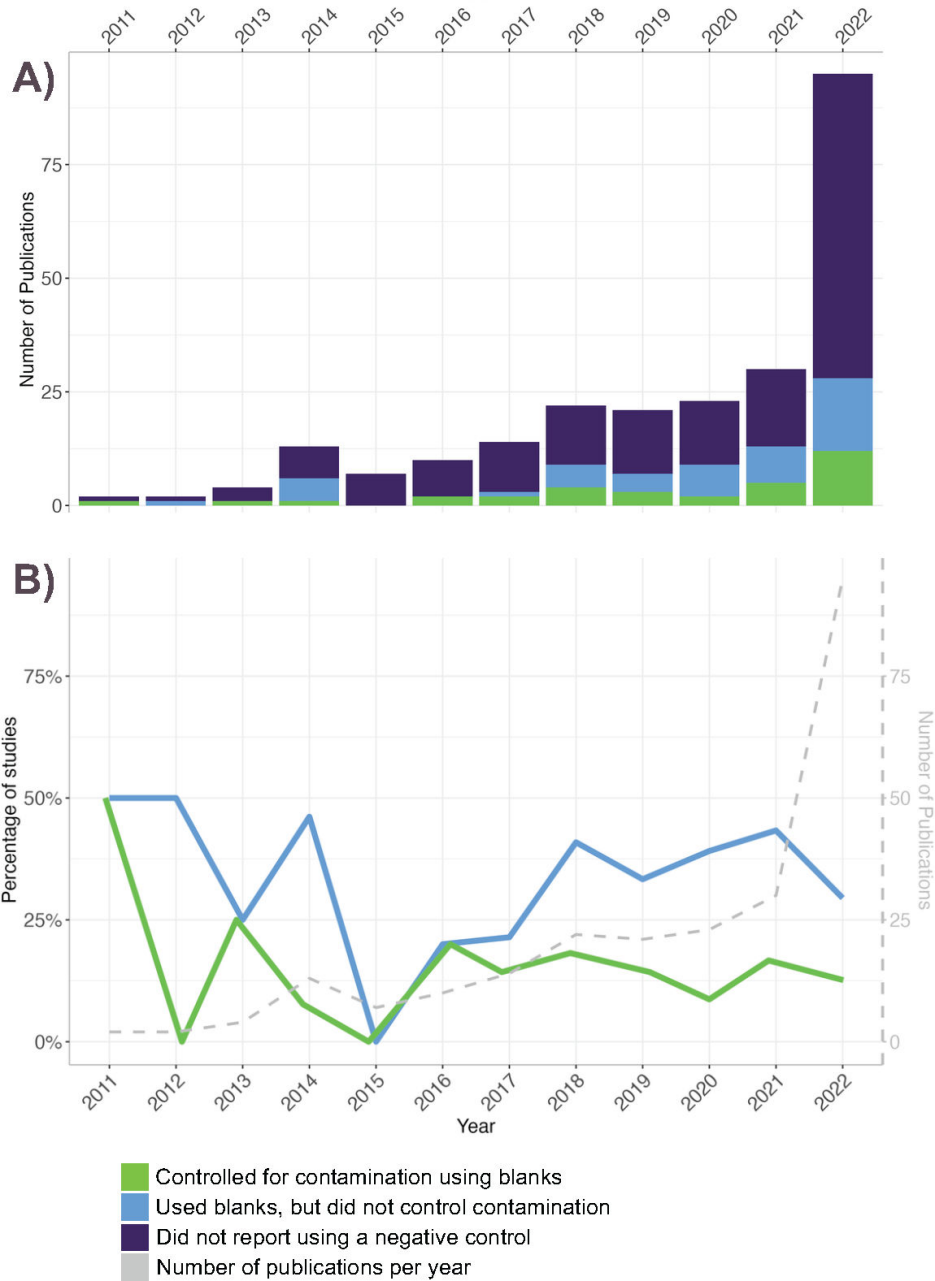
The trends in negative control use in our final pool of 243 papers from a 10 year period. (A) The number of studies published per year that included and sequenced negative controls to control for contamination (green), indicated the inclusion of negative controls but did not control contamination (blue), and those that did not include negative controls (purple). (B) The same data replotted as percentages of studies, with the total number of studies published per year shown in gray.

Only one-third (80/243) of studies reported using at least one negative control. Negative controls were referred to as blanks, extraction blank controls, no-template controls, PCR blanks, sequencing controls, or surface washes (from insects or tool rinses). Fewer than half (33/80) of the studies that reported including a negative control subsequently sequenced these and then compared the taxa found in their blanks to the corresponding insect samples. Therefore, only 13.6% of the studies included in the systematic review assessed their sequencing results for contaminating taxa. While the number of papers investigating insect microbiota increased exponentially between 2011 and 2022, the proportion of studies that used a negative control or controlled contamination per year has not increased (Fig. 3). Studies that did not control for contamination were not less frequently cited (*P* = 0.84), suggesting a lack of scrutiny when interpreting the conclusions derived from uncontrolled studies.

The low proportion of studies that report using negative controls (Fig. 3) signifies that a large part of insect microbiota research has thus far not adhered to the latest guidelines that are recommended to control for contamination (5, 8, 33). Although some studies may have used negative controls without mentioning them, this cannot be assumed.

The implication of this finding is that for 86.4% of studies included in our metadata, it cannot be conclusively said whether the microbiota reported are truly insect-associated or the byproduct of contamination. One of the challenges in recognizing contamination in insect studies is that some taxa that are commonly reported as insect microbes overlap with those of common contaminants. For example, *Pseudomonas* (34), *Acinetobacter* (35, 36), *Staphylococcus* (37), *Bacillus* (38), and *Burkholderia* (39) are all described insect-associated bacteria yet are also common contaminant taxa (5, 14, 19, 28).

Importantly, we also noted that there was considerable variation in the approaches used by the 33 studies that controlled for contamination. A total of 7/33 of studies used the statistical package Decontam (9) to call putative contaminant ASVs. The remaining studies used heuristic approaches, often involving a comparison between the communities identified or manually filtering prevalent taxa found in negative controls from samples. Sometimes, taxa were filtered based on the number of reads of putative contaminants from the controls, as a crude detection limit measure.

Without measuring the LoD, it is difficult to reliably distinguish true taxa from the contamination-induced “background noise” that is inevitably introduced during sampling and sequencing processes. Only 1.6% (n = 4 studies) measured the LoD using qPCR. The absence of measurements for the LoD, coupled with the few papers that controlled for contamination, further supports the finding that few authors accounted for potential contamination in their results. An alternative method to measure the absolute abundance and LoD is the inclusion of known amounts of artificial DNA or cells known as “spike-ins” (40). While not assessed in this systematic literature review, as long as the spike-in’s identity is carefully considered to ensure their absence in the target communities, they can offer some advantages over qPCR, including being cost-effective and providing quantification post-sequencing, accounting for the entire workflow including PCR amplification and sequencing biases.

### Acknowledging off-target amplification from plant/animal DNA

Reporting the number of reads from off-target DNA is highly recommended to provide an accurate overview of the sequences recovered, and to indicate the potential microbial biomass of samples and resulting sensitivity to introduced DNA contamination. We investigated what percentage of studies acknowledged the presence of chloroplast or mitochondrial DNA, the most predominant forms of off-target DNA in insect microbiota studies, and found that 35.2% included such acknowledgment. Most were studies that included a filtering step to remove chloroplast and/or mitochondrial DNA from their sequencing results, without necessarily disclose the proportion of nonmicrobial DNA in the data. Only 5.8% (n = 14 studies) of total studies quantified the amount of off-target amplification. It is possible that studies did not provide this information because off-target DNA represented few reads or was absent. However, disclosing the number of off-target reads can still be useful for comparing 16S data between insect microbiota studies.

In addition to microbial biomass, the amount of off-target DNA amplification also depends on the diet of the specimen, gut content at the time of sampling, and the primers used for PCR (20). Therefore, there can be considerable variation in the amount of off-target DNA and amplification bias within insect species depending on study design. The implication is that there can be differences in the bacterial abundances and diversity assessments, particularly in the representation of low-abundance taxa, between studies. In these cases, reporting the proportion of off-target DNA can indicate the extent of the potential amplification bias and facilitate comparison between 16S data sets.

### Concluding remarks and recommendations

The vast majority of studies included in our systematic review did not follow best practice for controlling contamination. Consequently, a large proportion of published insect microbiota may be contaminated, and this has the potential to mask and misrepresent true insect microbiota. While it is impossible to know the true number of studies affected, the lack of contamination control found in this systematic review suggests that it could be high. This reveals a strong need for improved methodological rigor and adherence to standard guidelines across insect microbiota research.

While this study focuses specifically on insect microbiota studies, the problem of contamination likely extends to other animal, plant, and environmental samples. Insects are not uniquely rife with contamination. All studies that use DNA sequencing are potentially susceptible to contamination, and low-biomass samples can be common—though often unrecognized—in many environments. Furthermore, we do not expect insect researchers to be uniquely likely (or not) to control for contamination. Hence, we speculate that a lack of contamination control is similarly widespread in other fields of research that use 16S sequencing.

To address contamination, we recommend the RIDE checklist, a minimum standards guide for low microbial biomass studies that can be easily integrated into study design (5). In addition to the four original guidelines, based on our findings, we propose including an additional guideline. The RIDES checklist stands for (1) Report methodology, (2) Include controls in sequencing, (3) Determine the level of contamination to measure the limit of detection, (4) Explore the impacts of contamination in downstream analysis, and (5) State the amount of off-target DNA amplification to disclose potential amplification bias.

This checklist can and should be paired with biologically informed inspections of the data. For example, bacteria that are found at consistent levels across species, habitats, tissues, or other factors that often differentiate microbiomes should raise suspicion as potential contaminants. The dominant taxa should also be carefully scrutinized. How plausible is it that they are true symbionts? Taxa characteristic of human skin, the ocean, or extreme environments should be treated with caution. Adopting these recommendations will improve the accuracy and standardization of future work and reduce the uncertainty posed by undisclosed amplification bias and uncontrolled DNA contamination in the field of insect microbiota research.

## Data Availability

All data on our filtering and metadata set are available in the supplementary materials. The code used to analyze the data is available on Github at https://github.com/hi-its-lisou/BlanksSLR.git.

## SUPPLEMENTAL MATERIAL

Figure S1 mbio.02658-24-s0001.pdf The empirical relationship between contamination and sample biomass.

Supplemental analysis mbio.02658-24-s0002.docx Additional considerations when assessing the nature of 16S sequences--transient bacteria and relic DNA.

SLR metadata mbio.02658-24-s0003.xlsx Metadata from the final pool of 243 insect microbiota studies.

Supplemental material mbio.02658-24-s0004.pdf Tables S1 and S2 and Figures S2 to S4.

ASM does not own the copyrights to Supplemental Material that may be linked to, or accessed through, an article. The authors have granted ASM a non-exclusive, world-wide license to publish the Supplemental Material files. Please contact the corresponding author directly for reuse.
