# Supplementary material for: Blanking on blanks: few insect microbiota studies control for contaminants
Source: mBio. 2025 Feb 25;16(4):e02658-24. doi: 10.1128/mbio.02658-24 (PMC11980574; doi:10.1128/mbio.02658-24)
Supplement: Figure S1 — The empirical relationship between contamination and sample biomass. [file mbio.02658-24-s0001.pdf]

Supplementary materials - Figure S1

The empirical relationship between contamination and sample biomass.

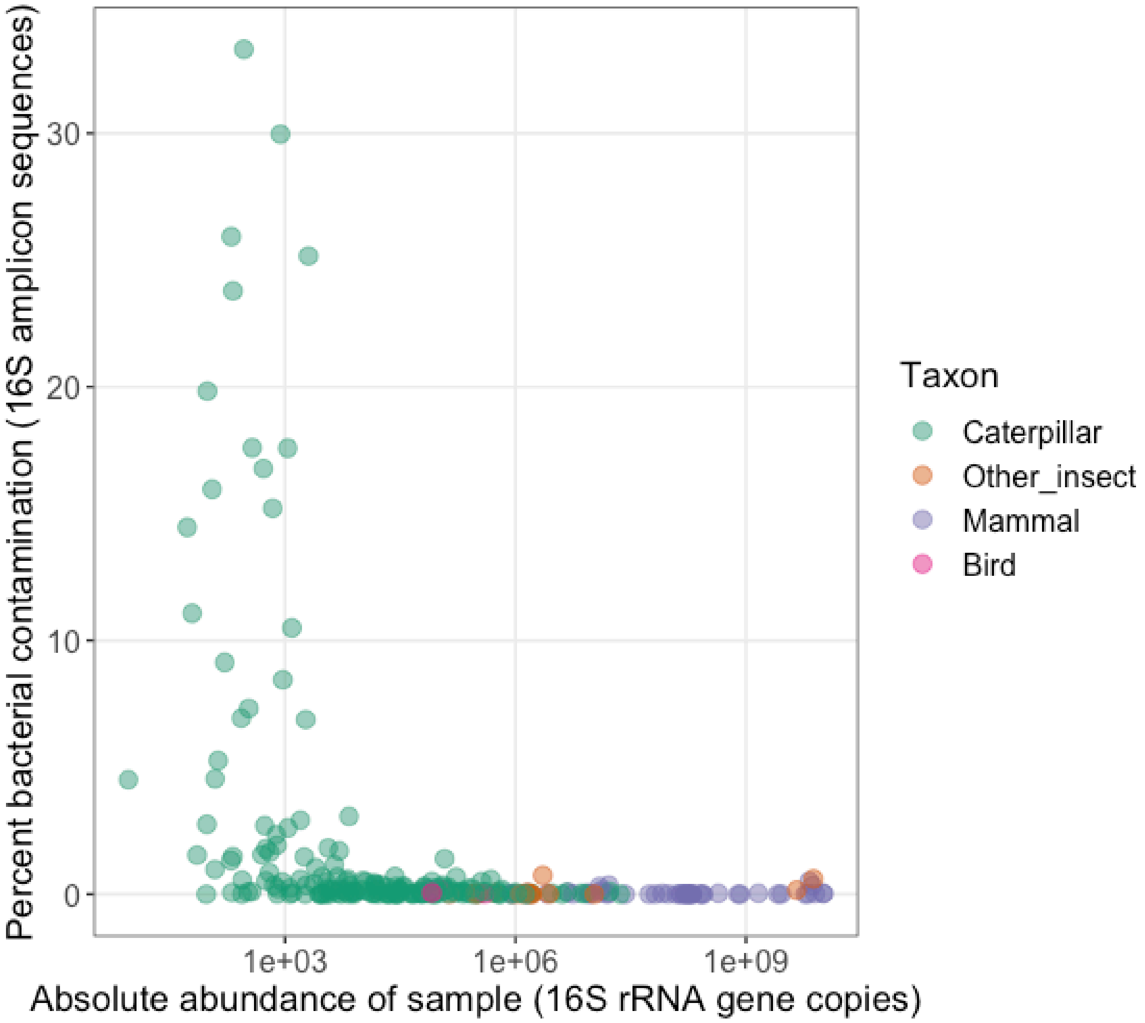

Figure S1: The empirical relationship between microbial biomass and contamination. Samples with varying levels of biomass are plotted against the percentage of bacterial contamination per sample. Low biomass samples are from caterpillars (green) moderate biomass samples are from other insects and birds (organs - pink), and high biomass samples are from mammals (purple).
