# Supplementary material for: Blanking on blanks: few insect microbiota studies control for contaminants
Source: mBio. 2025 Feb 25;16(4):e02658-24. doi: 10.1128/mbio.02658-24 (PMC11980574; doi:10.1128/mbio.02658-24)
Supplement: Supplemental analysis — Additional considerations when assessing the nature of 16S sequences--transient bacteria and relic DNA. [file mbio.02658-24-s0002.docx]

**Supplementary analysis: Additional considerations when assessing the nature of 16S sequences - Transient bacteria and Relic DNA**

There are many important factors to consider when using 16S sequencing to characterize insect microbiota, with the focus of this systematic review being to investigate how contamination is dealt with in the literature. However, DNA contamination isn’t the only factor that can influence assessments on taxonomic diversity and ecological function. Transient bacteria and relic DNA are also important considerations when interpreting sequencing results and assessing microbial assemblages.

While contamination, mitochondria and chloroplasts can be filtered based on taxonomy, transient microbes and relic DNA are much harder to distinguish, yet just as ubiquitous in the environment. Transient microbes do not replicate within a host, are often temporarily passing through, having positive, negative or negligible effects on insect hosts [1]. Relic DNA is the genetic material left behind from dead cells that can still be amplified and sequenced [2]. Both transients and relic DNA have the potential to influence ecological assessments of microbial communities as they interact differently with hosts compared to beneficial symbionts or pathogens [2, 3].

Despite this, only 22.5% of papers acknowledged transient bacteria and 3.6% acknowledged relic DNA as potential sources of DNA in their sequencing results. This finding suggests that some studies may be assuming symbionts, and overestimating their importance to the host. Without acknowledging alternative explanations, such as relic or transiency, we may be limiting our understanding of these complex ecological systems.

**References:**

1. Hammer TJ, Sanders JG, Fierer N. Not all animals need a microbiome. FEMS Microbiology Letters 2019;366(10)
2. Lennon JT, Muscarella ME, Placella SA, Lehmkuhl BK. How, When, and Where Relic DNA Affects Microbial Diversity. mBio 2018;9(3):10.1128/mbio.00637–18.
3. Snell Taylor SJ, Evans BS, White EP, Hurlbert AH. The prevalence and impact of transient species in ecological communities. Ecology 2018;99(8):1825–1835.
