# Supplementary material for: Blanking on blanks: few insect microbiota studies control for contaminants
Source: mBio. 2025 Feb 25;16(4):e02658-24. doi: 10.1128/mbio.02658-24 (PMC11980574; doi:10.1128/mbio.02658-24)
Supplement: Supplemental material — Tables S1 and S2 and Figures S2 to S4. [file mbio.02658-24-s0004.pdf]

Supplementary material -
Search strategy, filtering criteria, and metadata collection method.

Database advanced search inputs		
**Scopus**	**Web of science**	**Google Scholar**
ALL ("microbiome" OR "microbiota" OR "bacterial community" OR "bacterial communities" OR "microbial communities" OR "microbial community" OR "microbe" OR "bacteria") AND TITLE-ABS-KEY ("insect" OR "insects" AND NOT fish OR birds OR arthropods) AND ALL ("16S" OR "16S rRNA gene sequencing" OR "Next Generation DNA Sequencing" OR "High Throughput Nucleotide Sequencing" OR "Illumina Sequencing") AND PUBYEAR > 2010 AND PUBYEAR < 2023 AND (LIMIT-TO (DOCTYPE , "ar") OR LIMIT-TO (DOCTYPE , "dp") OR LIMIT-TO (DOCTYPE , "English"))	TS=(insect OR insects OR entomology) AND ALL=(microbiome OR microbiota OR bacterial community OR bacterial communities OR microbial communities OR microbial community OR microbe OR bacteria) AND ALL=(16S OR 16S rRNA gene sequencing OR Next Generation DNA Sequencing OR High Throughput Nucleotide Sequencing OR Illumina Sequencing) AND DT=(Article OR Data Paper) AND LA=(English) AND PY=(2011-2022)	insect OR insects AND microbiome OR microbiota OR bacterial community OR microbial community AND 16S rRNA gene sequencing OR Next Generation DNA Sequencing
**Number of hit results: 2937**	**Number of hit results: 1215**	**Number of hit results: 17000**

Figure S2: The advanced search input used for Scopus, Web of Science, and Google Scholar. The search results were ordered by relevance, and the first 200 studies were extracted and uploaded to Covidence software to begin filterering.

Table S1: The inclusion and exclusion criteria used for advanced searches and manual screening. The final pool of papers strictly adheres to these requirements.

Criterium	Include	Exclude
Language:	English	Other
Years:	2011 - 2022	Other
Article type(s):	Peer-reviewed journal articles and data papers	Review, opinion, survey, thesis, or book
Subject type(s):	Insects (i.e., Class - Insecta)	Exclusively or mainly other animals, insect food, nest or environmental microbiota
Target microbiome(s):	Gut, whole insects, body segments, exoskeleton, glands using universal 16S primers	Exclusively fungal internal transcribed spacer (ITs), viruses, or specific 16s primer
Data output:	16S community analysis, primary dataset	For studies that use the same or overlapping datasets, the multiples are excluded to only include one
Sequencing method:	Next-generation/High throughput/Illumina sequencing, and 454 pyrosequencing	Culture dependent, Cloning, PCR-DGGE, TRFLP, bTEFAP, PCR-TTGE, or sanger sequencing

Exclusion Annotations

- Non-insect (microbiome descriptions for spiders, ticks, plants, soil, chickens, pigs, human, cadavers, genomic analyses, wine, annelids, or frass)
- No 16s (no original 16s results, no 16s in methodology, reviews, current opinions)
- Cultures (isolates, culture dependent, strain specific)
- Specific primers
- Cloning
- PCR DGGE
- Insect food (not including the insect itself)
- bTEFAP
- TRFLP
- Not English

Figure S3: The list of justifications given to a study when it was filtered/excluded from our final pool of studies for metadata extraction.

Table S2: The data extracted from our final pool of studies, and details on how/where the information was obtained from.

Metadata Extraction<br>Information Gathered and Search Methods			
**Metadata**		**Information gathered**	**Search method**
**Background info**	**Title**	[Title of the study]	Exported from databases, imported into Covidence
	**Year**	[Year of publication]	
	**Authors**	[Authors of the study]	
	**Journal published in**	[Journal where the study was published]	
**Research subject**	**Order of insect**	[Order of insect used in the study]	From methods section
	**Family of insect**	[Family of insect used in the study]	
	**Development stage(s) used in study**	• 'Adults only'<br>• 'Juveniles only'<br>• '>1 development stage'	
	**Target community of study**	• 'Gut'<br>• 'Body' (whole insect)<br>• 'Other' (specific glands/organs, cuticular, or both internal and external communities targeted)	
	**16S rRNA gene target region**	[Region covered based on the primers used] (cross-referenced with Abellan-Schneyder et al. 2021).	
**Sampling and extraction method**	**Sampled**	• 'Gut'<br>• 'Whole'<br>• 'Other' (sample both dissected guts and whole insects, washes, or specific glands)	From methods section
	**DNA extraction kit used**	[Manufacturer of DNA extraction kit used, or 'Protocol' if no kit used]	
	**Surface sterilisation**	• 'No'<br>• 'Yes'	

Metadata		Information gathered	Search method
**Use of negative controls**	**Did they use a negative control?**	• ‘No’<br>• ‘Yes’<br>↳ [What kind?]<br>↳ [How many?]<br>→ Do they sequence them?<br>• ‘No’<br>• ‘Yes’<br>→ Did they control contamination using them?<br>• ‘No’<br>• ‘Yes’<br>↳ [Method used]	Keywords search - “negative”, “control”, “blank”, “extract”, “contam”
**Quantification**	**Do they use qPCRs in tandem?**	• ‘No’<br>• ‘Yes’<br>→ Do they determine the limit of detection?<br>• ‘No’<br>• ‘Yes’	Keywords search - “quanti-”, “qPCR”
**Alternative sources of DNA**	**Animal/plant DNA**	• ‘No’<br>• ‘Yes’ (must be acknowledged in methods, results, or discussion)	Keywords search - “mito-”, “chloro-”, “plant”, “animal”
	**Transiency**	• ‘No’<br>• ‘Yes’	Keywords search - “transi-”, “environm-”
	**Relic DNA**	• ‘No’<br>• ‘Yes’	Keywords search - “relic”, “dead”, “remnant”, “residual”
**Data availability**	**Do they provide a project or accession number for sequence data?**	• ‘No’<br>• ‘Yes’	Keywords search - “proj””, “acc-”
**Citations**	**How many citations do they have?**	[Number of citations]	Crossref API via the rcrossref package in R

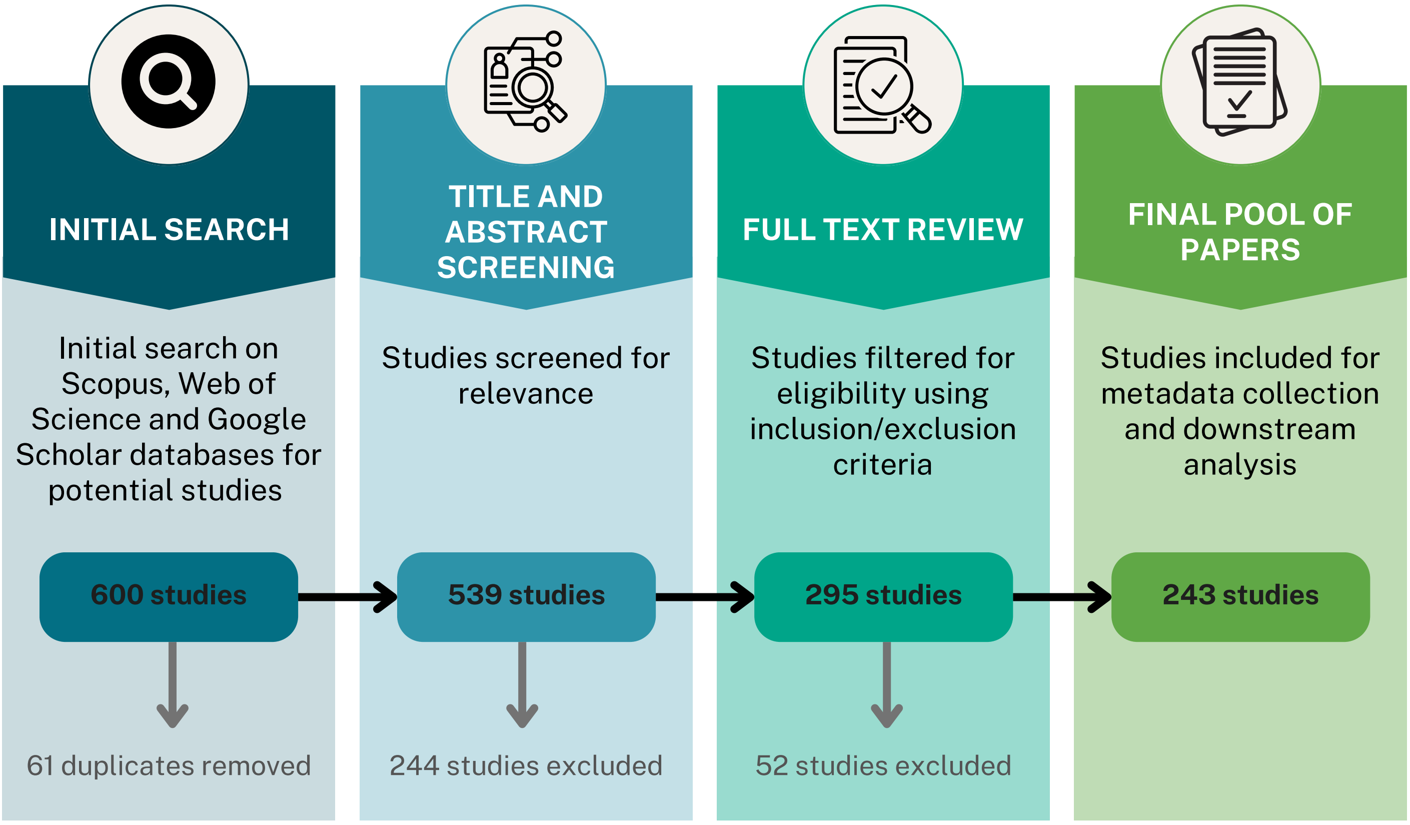

Figure S4: The number of papers that passed each filtering step, with the final pool of papers (n=243) being used for metadata collection
